# The gender-affirming model of care is incompatible with competent, ethical medical practice

**DOI:** 10.1177/10398562241239478

**Published:** 2024-03-19

**Authors:** Andrew Amos

**Affiliations:** Division of Tropical Health and Medicine, College of Medicine and Dentistry, 104397 James Cook University, Townsville, QLD, Australia

**Keywords:** gender dysphoria, medical ethics, phenomenology, psychopathology

## Abstract

**Objective:**

To examine the compatibility of gender-affirming care with the principles and practices of psychiatry.

**Conclusions:**

The assumption that there is no pathology involved in the development of gender diversity is a necessary precondition for the unquestioning affirmation of self-reported gender identity. Cases where psychosis is the undeniable cause of gender diversity demonstrate this assumption is categorically false. To protect this false assumption, gender-affirming guidelines forbid the application of the core psychiatric competencies of phenomenology and psychopathology to the assessment of gender diversity. They substitute the political goal of expanding personal liberty for the evidence-based medicine processes of clinical reasoning, rendering them incompatible with competent, ethical medical practice.

Given the almost complete lack of high-quality evidence regarding the nature and treatment of the experiences currently clustered under the title gender diversity,^1–4^ the rapid increase in presentations and the resources allocated to them in Australia is remarkable.^5,6^ Clinical guidelines describing the dominant treatment paradigm for gender diverse patients, the gender-affirming model of care (GAMOC), assert without evidence that pathology plays no part in the development of gender diversity.^1,7^

## The evolution of models of gender diversity

The nature of gender diversity is unclear because the terms and concepts used to understand it continually change.^1,7^ Drescher^8^ summarised the history, starting with the mid-20^th^ century diagnosis transsexualism, a form of sexual deviance associated with homosexuality, defined as living as a member of the opposite to one’s biological sex. The subsequent variety of presentations indicated that sexuality and preferred gender were substantially independent, leading to the replacement of transsexualism by the diagnosis gender identity disorder in the late 1980s.

The gender diverse community welcomed the separation of gender identity from sexual deviance, alongside the reconceptualisation of homosexuality itself as a healthy form of human behaviour.^9^ However, many interpreted the introduction of gender identity disorders into the DSM-III and ICD-10 as a pathologization of their sense of self.^10^ This triggered the depathologization movement which continues to apply pressure to the American Psychiatric Association and World Health Organization to remove all gender diversity diagnoses from the DSM and ICD.^10^ The movement was instrumental in changing gender identity disorder to gender dysphoria in DSM-5, and categorising gender incongruence as a form of sexual health condition rather than a mental disorder in ICD-11.^8,11^ The influence of activists is concerning given that both categories are based on clinician consensus rather than empirical evidence, due to the small number of patients involved.

## The circular assumptions of gender-affirming care

The driving principle of the GAMOC is that health care professionals cannot assess but must affirm patient-reported gender identity.^1,12^ The emergence of non-binary and fluid genders means there are no boundaries to self-reported gender identity, which may include a gender consistent with one of the two biological sexes; a combination of features consistent with both sexes; the absence of features of gender; an identity as a voluntarily/involuntarily castrated eunuch; or arbitrary and rapidly changing variations.^1,7,13,14^

The principle of unquestioning affirmation of gender identity critically relies upon the assumption that pathology plays no part in the development of gender diversity. If it is admitted there are some pathological causes of gender diversity, then it becomes necessary to assess the health or illness of all presentations. Despite the existential reliance of the GAMOC upon this assumption, it has never been tested, or even questioned, by GAMOC advocates.

The World Professional Association for Transgender Health (WPATH) endorses the leading international standards of care for treatment of gender diverse patients. They assert that ‘[g]ender diversity is a natural variation in people and is not inherently pathological’ (pS34). However, no evidence is presented and the supporting reference leads back through the previous version of the Guidelines to a statement by the WPATH Board of Directors.^4,15^ Not only do the guidelines rely on a circular reference to an evidence-free assertion of this core assumption of their model, GAMOC advocates reject the possibility of testing the model using randomised control trials as unethical.^16^

## The phenomenology and psychopathology of gender diversity

From a psychiatric perspective, the proposition that psychopathology plays no role in gender diversity is absurd. The most detailed personal description of the experiences of psychosis is that of Daniel Paul Schreber, a German judge who minutely described his belief that God had turned him into a woman and was sending ribbons from the sun through his body to impregnate him and repopulate the earth.^17^ It is difficult to imagine a more pathological aetiology for gender diversity, yet the GAMOC provides no framework for assessing such a patient, and does not view Schreber’s case as an absolute contraindication to social, medical, or surgical transition.^1,4,18^

While GAMOC advocates have argued transition is safe in patients with psychosis because it is easy to differentiate psychotic from non-psychotic aetiologies of gender diversity,^4^ they have provided no guidance on how to do so, and no empirical evidence that it is safe to try. To the extent they discuss the role of psychosis or severe personality pathology in the development of gender diversity at all it is only to deny that either might prevent transition.^1,4,7,18^

The WPATH standards acknowledge the small evidence base on differentiating psychotic from non-psychotic aetiologies of gender diversity, comprised entirely of case reports. Their main reference on the topic noted that of 19 previously published cases 16 had been judged psychotic in the absence of gender dysphoria, with 4 of these nonetheless treated with hormones or surgery and suffering harm as a result.^18^ Another review indicated that up to 6% of patients with gender dysphoria had a comorbid psychotic disorder, and listed a number of case studies where antipsychotic treatment was associated with a reduction or resolution of gender dysphoria.^19^ Despite this, the WPATH standards appear more concerned that comorbid psychosis might prevent gender diverse patients from accessing the GAMOC than that patients with psychosis might be harmed by the affirmation of psychotic beliefs.^1^

Close reading of GAMOC guidelines reveals a fatal deficiency. The guidelines assert that the experience of a gender identity that is different from biological sex is in all cases a healthy variant of normal,^1,7^ but they do nothing to explain the nature or variance of the experience of gender identity, what it means for gender identity to be different from biological sex, or healthy and pathological variations. This complete failure to describe the phenomenology and psychopathology of gender diversity makes it impossible for the guidelines to meaningfully describe what gender diversity is, or to demonstrate that it does not involve pathology.

## The politics of personal liberty and the abandonment of clinical responsibility

Gender identity is a concept describing a type of human experience. It can only be understood by applying the clinical skills of psychiatry with knowledge of phenomenology. As Schreber illustrates, it is certain that pathology causes some cases of gender diversity. Differentiating between healthy and pathological gender diversity, or, more likely, gauging the relative contribution of healthy and pathological processes originating within or in the environment of each patient, can only be achieved by the comparison of an individual’s patterns of behaviour with patterns of normal and pathological development.

Phenomenology and psychopathology are core competencies of psychiatric practice, and of no other medical specialty, yet the GAMOC guidelines are designed to exclude psychiatric skills and knowledge. As should be clear from the foregoing discussion, the reason is that the GAMOC’s core clinical principle of unquestioning gender-affirmation, and the core assumption on which it relies – that gender diversity by definition is never caused by endogenous pathology – are both incompatible with competent and ethical psychiatric practice.

Thus, it is misleading to think of the GAMOC guidelines as primarily clinical documents. In place of medical diagnosis, they assert a political right designed to expand the boundaries of personal liberty: the right to define a gender identity. In the current formulation of the GAMOC, this is an absolute right with no fixed definition and no constraints.^1,7^ Self-defined gender identity does not have to be coherent, persistent, or intelligible to a healthcare provider or the average citizen.^12^ Traditional guidelines, such as the RANZCP guidelines for the treatment of mood disorder,^20^ outline a process of clinical reasoning which matches diagnoses to treatments informed by patient preferences based on risk-benefit analyses. GAMOC guidelines abandon the clinical discipline of diagnosis and make treatment contingent upon the unconstrained subjective experiences of children and potentially disturbed adults. This is unethical, because modern medicine relies upon accurate diagnosis and evidence-based clinical reasoning to ensure that treatment is likely to help and not harm patients.

The depathologization movement raises homosexuality as a model of the potential social goods and lack of harms that can be achieved by eliminating a stigmatising diagnosis. However, as Meyer^21^ points out, homosexuality was only redefined after a debate where ‘we as a society and as scientists agree [on what] are abnormal behaviours, cognitions, and emotions’ allowing for the emergence of ‘a scientific and social consensus’ (p675). No such debate has been started, and no such consensus yet exists for gender identity.

Gender-affirming care is fundamentally incompatible with competent, ethical medical practice. It predicates a class of experiences which diverge from those of the vast majority of human beings, but refuses to describe normal experience or the patterns of divergence. It assumes there are no pathological aetiologies of gender diversity and protects this assumption by forbidding the assessment of pathology in individual patients, and by forbidding the evaluation of treatment outcomes by RCTs.

## The RANZCP position on the GAMOC

While the RANZCP initially endorsed the GAMOC, in Position Statement 103 (PS103), it removed this endorsement without explanation,^22^ indicating that while some patients prefer affirmation, the evidence about the benefits and harms of providing or withholding GAMOC does not justify its recommendations. PS103 does not provide any evidence or rationale for its statement that ‘Being Trans or Gender Diverse does not represent a mental health condition’.^23^ In essence, the RANZCP advises psychiatrists to be aware that the GAMOC exists, but to provide appropriate patient-centred, evidence-based psychiatric care for mental health conditions as if it did not.

This appears to be a pragmatic compromise that allows PS103 to avoid a more critical position on the GAMOC by limiting its scope to the treatment of mental illness. Apart from the untested and otherwise undeveloped assertion that gender diversity ‘does not represent a mental health condition’, PS103 is entirely consistent with the arguments made above. Although it is clear that this compromise balances the concerns of different stakeholders, the medicolegal implications for psychiatrists and their patients may be too important to long defer a conclusive position on the aetiological role of mental illness in gender diversity. For example, the lack of evidence for the GAMOC has led one insurer to restrict reimbursement for private practitioners treating gender dysphoria.^24^ In addition, the courts have relied upon medical college positions to assume that GAMOC is the accepted standard of care for gender diversity in Australia.^22^ Given these stakes, the RANZCP should either provide the evidence and rationale for the position that mental illness plays no aetiological role in gender diversity, or acknowledge that it does play a role in some or all cases and facilitate the phenomenological and psychopathological understanding necessary for safe and ethical treatment.

## Conclusions

In the absence of models of the phenomenology and psychopathology of gender diversity, it is impossible to meaningfully judge what proportion of cases involves pathology or assess the role of pathology in individual patients. Unquestioning gender-affirming care is therefore unable to exclude the possibility that it is reinforcing the pathologies of some, most, or all of its patients. This is unethical, and it is the responsibility of psychiatrists to ensure that no patients are harmed by this dangerous model of care.
